# An International Classification of Functioning, Disability and Health Model-Based Analysis of Suicidal Ideation among 9920 Community-Dwelling Korean Older Adults

**DOI:** 10.3390/healthcare12050538

**Published:** 2024-02-23

**Authors:** Haewon Byeon

**Affiliations:** Department of Digital Anti-Aging Healthcare (BK21), Inje University, Gimhae 50834, Republic of Korea; bhwpuma@naver.com; Tel.: +82-10-7404-6969

**Keywords:** older adults, suicidal ideation, depression, International Classification of Functioning, disability

## Abstract

Background: Many complex factors contribute to suicide in older adults. The suicidal ideation that precedes suicide is an especially direct predictor of suicide. This study aimed to identify the effects between variables affecting suicidal ideation among older adults using the International Classification of Functioning, Disability and Health (ICF) model and understand the causal relationships to systematize complex factors. Methods: This study used data from 9920 community-dwelling older adults who completed a national survey in 2020 to classify predictors of suicidal ideation (e.g., depression, subjective health status, sociodemographic factors, health factors, social support, instrumental activities of daily living (IADL), and social participation) by using the ICF model. To determine the causal relationship between variables, this study examined significance based on the critical ratio (C.R.) and squared multiple correlation (SMC) by using a path model. Results: Gender, education level, economic level, age, IADL, relationship satisfaction with a child, depression, and the number of chronic diseases significantly affected suicidal ideation, while age, employment status, participation in social groups, formal and informal support, satisfaction with a friend/neighbor relationship, and subjective health status did not significantly influence it. Moreover, depression mediated the relationship between each of these variables and suicidal ideation. Conclusions: It was found that depression was the most direct and mediating factor in suicidal ideation among many factors affecting the suicidal ideation of community-dwelling older adults. Additional studies should be conducted to develop community-level strategies based on these factors and understand causal relationships.

## 1. Introduction

Among OECD member countries, South Korea has had the highest prevalence of depression for 18 years [1] and the highest suicide rate for the past 20 years, except for 2017 [2]. The suicide rate of the Republic of Korea is 24.6 per 100,000 people, more than twice the mean suicide rate of OECD member countries (11.0 per 100,000 people) [3]. The same trend can be found among older adults: the suicide rate of South Koreans in their 60s (33.7 per 100,000 people), that of South Koreans in their 70s (42.6 per 100,000 people), and that of South Koreans in their 80s or older (67.4 per 100,000 people) was 2.2, 2.8, and 3.1 times higher than the mean suicide rates of OECD member countries, respectively [3]. Among older adults, the number of suicides increases with age. The suicides of older adults account for more than 30% of all suicides.

Suicide is a series of processes, including suicidal ideation, suicide planning, and suicide attempts [4,5]. It is a continuous concept that cannot be broken down into distinct stages, rather than a single concept [4,5]. Suicidal ideation is a direct predictor of suicide and has important healthcare implications [6,7]. Systematic reviews and meta-analysis studies reported that gender, age, psychological factors (e.g., stress), physical and mental illness, economic hardship, emotional reasons (e.g., loneliness and isolation), and decreased social support were the factors affecting suicide among older adults [8,9,10]. These findings suggested that a range of factors, including environmental and economic factors, could contribute to suicide in older adults and implied that these factors interacted [11].

Recent studies have further elucidated the multifaceted nature of suicide risk among older adults, highlighting the significance of both individual and societal factors. Cabello et al., (2020) [12] reported that higher negative affect and higher disability were associated with 12-month suicidal ideation and suicide attempts for both young and middle-aged adults and older adults, indicating that these factors transcend age groups in their impact on suicide risk. Additionally, Kim and Kwon (2012) [13] examined the risk factors for suicidal ideation and suicide attempts among Korean older adults, finding that decreased health-related quality of life (HRQoL) strongly affected suicidal ideation, regardless of disease status, underscoring the importance of HRQoL in suicide prevention efforts. Kim and Kwon’s study (2012) [13] on depressed adults identified specific factors influencing suicidal thoughts, such as income, number of family members, marital status, subjective health, stress, economic activity status, diabetes, alcohol consumption, and Body Mass Index (BMI), suggesting that interventions to prevent suicide should consider these diverse influences.

**Figure 1:**
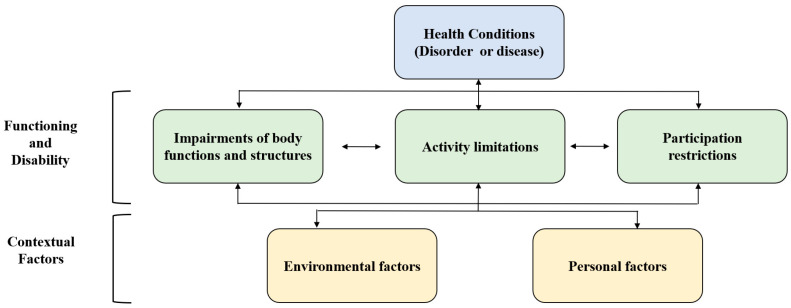
Components of the ICF [14].

The International Classification of Functioning, Disability and Health (ICF) has been widely used in recent years as a suitable concept for explaining various factors regarding health status from a complex perspective. The ICF, developed by the World Health Organization, is an international classification related to health. The ICF is a conceptual framework that can be applied to management related to an individual’s health by establishing social factors. The ICF consists of two factors (functional performance and impairment, and background) and five elements (impairments of body functions and structures, activity, participation, environmental element, and personal element) to describe health status (Figure 1) [14].

Recent applications of the ICF framework have further demonstrated its utility in assessing and managing health status across diverse populations and conditions. Hwang et al., (2023) [15] explored the impact of appetite alteration on self-management and malnutrition in maintenance hemodialysis patients, utilizing the ICF framework to validate an appetite alteration model. This study highlighted the framework’s ability to integrate qualitative and quantitative data to address complex health issues. Rautiainen et al., (2023) [16] proposed a model for monitoring individual health status by calculating a personal health index based on the ICF framework, demonstrating its applicability in handling incomplete and heterogeneous datasets. Kostanjsek (2011) [17] provided an overview of the ICF taxonomy and its use at both population and clinical levels, emphasizing the framework’s role as a common language for disability statistics and health information systems. Bufka, Stewart, and Stark (2008) [18] discussed the emergence of the ICF as the principal framework for describing health and health-related status, underscoring its significance in the field of health assessment. Despite this trend in the application of the ICF to disease, there is a paucity of studies analyzing the ICF model in relation to suicidal ideation using large, representative, epidemiological samples of the elderly population.

This study applied the ICF model to explain the variables reported as predictors of suicidal ideation in community-dwelling older adults from a composite perspective. This study used a path model to identify the effect of each variable on the suicidal ideation of community-dwelling older adults and discover their causal relationships.

## 2. Materials and Methods

### 2.1. Research Model

**Figure 2:**
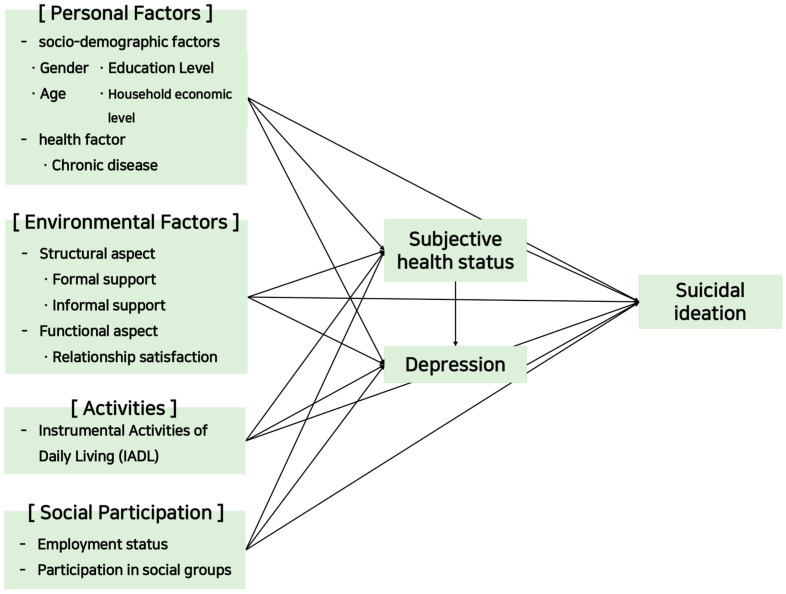
Research model.

This study set up a research model (Figure 2) to explore the relationships between relevant variables affecting suicidal ideation in community-dwelling older adults after the COVID-19 pandemic by focusing on subjective health status and depression, variables related to impairments of body functions and structures in the ICF model’s elements.

### 2.2. Data Source

This study used data from the 2020 National Survey of Older Koreans conducted by the Korea Institute for Health and Social Affairs and the Ministry of Health and Welfare. This study was approved by the Institutional Review Board (IRB) of the National Institute of Health and Social Research (no. 2020-36; 3 February 2020). According to the Declaration of Helsinki and its later amendment, 1964, all patients’ data were handled anonymously to maintain the confidentiality of the patients. The study was conducted according to the transparent reporting of an observational cohort study (STROBE). The National Survey of Older Koreans is a national statistic, conducted every three years to provide baseline data for establishing policies by identifying the living conditions, characteristics, and needs of older adults and changes in their characteristics. The 2020 National Survey of Older Koreans was conducted on 10,097 older adults (≥65 years old) living in general residential zones in 17 cities and provinces (969 survey districts) nationwide by using a stratified cluster sampling method [19]. Trained surveyors visited the sample households and conducted in-person interviews based on a designed survey table between 14 September and 20 November 2020 [13]. This study utilized data from 9920 older adults out of the 10,097 older adults who responded to the 2020 National Survey of Older Koreans after excluding those who could not measure items related to social support because they did not have a surviving child or grandchild.

### 2.3. Measurement and Definition of Variables

The variables used in this study were classified based on the ICF model. Suicidal ideation was measured as the experience of suicidal ideation after the age of 60 (yes or no).

Impairments of body functions and structures (subjective health status and depression): Subjective health status was defined as own usual health status, as perceived by an older adult (poor, fair, or good). In this study, referencing research such as that of Byeon and Been (2023) [20], depression was defined as an emotional functional impairment within the category of body functions. Depression was measured using the Korean version of the short form of the Geriatric Depression Scale (SGDS-K). A respondent answers each of the 15 items with ‘yes’ or ‘no’ (1 or 0, respectively), so the total score ranges between 0 and 15 points [14]. This study defined 8–15 points as depressive symptoms [19]. The Cronbach’s alpha of the SGDS-K was 0.8585 [21], and the Cronbach’s alpha in this study was 0.90.

Personal element (sociodemographic factors and health factor): The personal element was divided into sociodemographic factors and health factors. Sociodemographic factors included gender (male or female), age (65–74, 75–84, or ≥85 years old), education level (elementary school graduate or below, middle school graduate, high school graduate, or college graduate or above), and household economic level (national basic livelihood security recipient or not national basic livelihood security recipient), used as variables.

Environmental element (social support): Social support was divided into formal and informal support from the structural aspect, and functional support, indicating relationship satisfaction with a child and with a friend/neighbor. Formal support was measured by the use of a community senior citizen center or senior welfare center in the past year (yes or no), while information support was measured by the frequency (fewer than twice a year, once or twice every three months, once or twice a month, once a week, and almost every day) of meeting with an acquaintance (a child/child’s spouse/grandchild/sibling/relative/friend/neighbor not living together) in the past year. The functional aspect was measured by relationship satisfaction with a child and that with a friend/neighbor (satisfactory, fair, or unsatisfactory). The number of chronic diseases (1 or fewer, 2–4, or 5 or more) was a variable of the health factor. This was a personal factor because it was hard to change at the point of analysis.

Activity (instrumental activities of daily living): Instrumental activities of daily living (IADL) were measured with the Korean Instrumental Activities of Daily Living (K-IADL) [22]. The K-IADL consists of 10 items: grooming, housework, meal preparation, laundry, taking a prescribed dose of medicine on time, money management, a short-range outing, making purchases and decisions and paying and getting change, making and receiving phone calls, and using transportation. Among them, grooming, housework, meal preparation, laundry, taking a prescribed dose of medicine on time, money management, and a short-range outing were measured as fully independent (1 point), partially dependent (2 points), or fully dependent (3 points). Making purchases and decisions and paying and getting change, making and receiving phone calls, and using transportation were measured as fully independent (1 point), little dependent (2 points), much dependent (3 points), or fully dependent (4 points). However, these were re-categorized as fully independent = 1, partially dependent = 2, and fully dependent = 3. All measurement values were reverse-coded so that a higher score could indicate full independence. An IADL score of 25 points or lower was defined as difficulties in IADL, while a score of 26 points or higher was defined as no difficulties in IADL. The Cronbach’s alpha for the K-IADL was 0.94 [23], and the Cronbach’s alpha of this study was 0.88.

Social Participation (employment status and participation in social groups): Social Participation was a variable for social engagement among older adults, defined as employment and participation in social groups (yes or no).

## 3. Results

### 3.1. General Characteristics

Table 1 shows the general characteristics of older adults with suicidal ideation and those of older adults without suicidal ideation. Among 9920 subjects, 187 (1.9%) had suicidal ideation, while 9733 (98.1%) did not have suicidal ideation. These two groups generally had similar characteristics, but there were some differences. The prevalence of depression in older adults without suicidal ideation was 12.3%, while that of older adults with suicidal ideation was 53.3%. Moreover, for the group with suicidal ideation ratio, subjective health was reported to be twice as poor or more than the group without suicidal ideation. In addition, 3.5 and 5.8% of those without suicidal ideation were unsatisfied with their social relationships (relationships with a child and a friend/neighbor, respectively), while 20.3 and 24.6%, respectively, of those with suicidal ideation were unsatisfied with their social relationships.

### 3.2. Predictive Model Validation: Fitness of the Predictive Model

The structural equation modeling (SEM) conducted using AMOS provided a comprehensive evaluation of the predictive model’s fit with the observed data. The goodness-of-fit indices obtained from the analysis were as follows: Chi-square/degrees of freedom ratio (CMIN/DF) = 2.811, Goodness-of-Fit Index (GFI) = 1.000, Adjusted Goodness-of-Fit Index (AGFI) = 0.995, Comparative Fit Index (CFI) = 0.999, Tucker–Lewis Index (TLI) = 0.989, Root Mean Square Error of Approximation (RMSEA) = 0.014, and Standardized Root Mean Square Residual (SRMR) = 0.004. These indices collectively suggest that the model exhibits an excellent fit with the empirical data. The fitness of the predictive model is presented in Table 2.

### 3.3. Estimation of Path Coefficients of the Predictive Model

**Figure 3:**
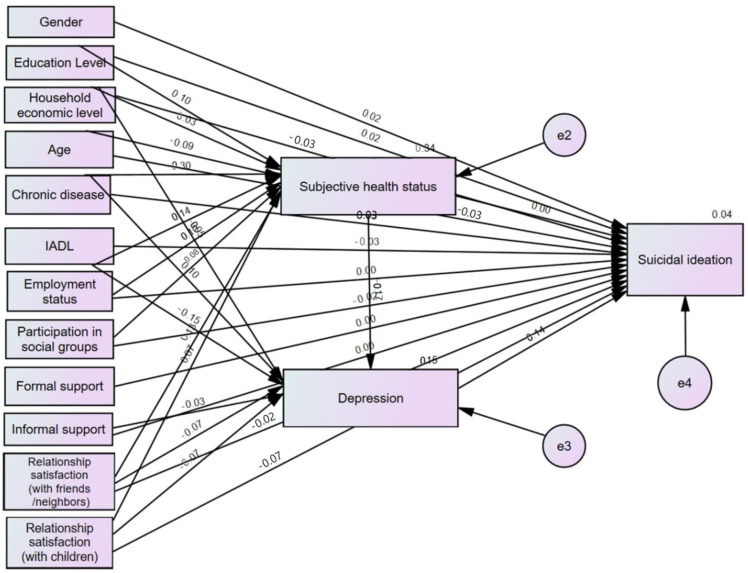
Standardized coefficients of the predictive model.

The analysis results of the prediction model of this study are presented in Table 3 and Figure 3, and the items presented are in the order of β value, standard error (S.E.), critical ratio (C.R.), and squared multiple correlation (SMC). Subjective health status was better with a higher education level, for non-recipients of national basic livelihood security, and for those with a younger age, fewer chronic diseases, higher IADL, employment status, higher informal support, and higher social support satisfaction (a child and a friend/neighbor), and they explained 33.8% of the variance.

It was found that respondents were more likely to experience depression when their subjective health was poorer, they received national basic livelihood security, they had more chronic diseases, they had lower IADL, their informal support was worse, and their satisfaction with social support (a child and a friend/neighbor) was lower. These variables explained 14.6% of the variance.

The results showed that respondents were more likely to have suicidal ideation when they were females, they had a higher education level, they received national basic livelihood security, they were younger, they had a lower IADL score, they had lower social support satisfaction with a child, they had depression, or they had more chronic diseases. These variables explained 3.9% of the variance.

### 3.4. Effect Analysis of the Predictive Model

Table 4 presents the total effect, direct effect, and indirect effect of this study. The variables affecting subjective health status were education level, household economic level, age, the number of chronic diseases, IADL, employment status, and satisfaction with social support (a child and a friend/neighbor).

Subjective health status, household economic level, the number of chronic diseases, IADL, informal support, and satisfaction with social support (a child and a friend/neighbor) were variables influencing depression. Among them, the total, direct, and indirect effects of household economic level, the number of chronic diseases, IADL, and satisfaction with social support (a child and a friend/neighbor) were significant, while only the total and indirect effects of subjective health status and informal support were significant.

It was found that gender, education level, household economic status, age, IADL, employment status, informal support, satisfaction with social support (a child and a friend/neighbor), subjective health status, depression, and the number of chronic diseases affected suicidal ideation. Among them, the total, direct, and indirect effects of household economic level, relationship satisfaction with a child, and the number of chronic diseases were significant. Moreover, the total and direct effects of gender, age, and depression and the total and indirect effects of IADL and relationship satisfaction with a friend/neighbor were significant. In addition, only the direct effects of education level and employment status were significant, and only the indirect effects of informal support and subjective health were significant. All indirect effects except for the subjective health status–suicidal ideation path were significant (Table 5).

## 4. Discussion

The results of this study showed that depression not only had the most direct effect on suicidal ideation but also played a mediating role between the variables in the ICF model and suicidal ideation. Previous studies support the intricate relationship between depression, suicidal ideation, and various psychosocial factors. For instance, Choi et al., (2022) [24] explored the effects of depression and suicidal ideation on the flourishing of high school students, revealing a moderated mediation model where a growth mindset played a crucial role in mitigating the impact of depression and suicidal ideation on students’ well-being. Similarly, Zhang et al., (2022) [25] found that impulsiveness indirectly affects suicidal ideation through depression, emphasizing the moderating role of impulsiveness in the relationship between depression and suicidal ideation. Lastly, Fu et al., (2023) [26] assessed the mechanisms between HIV-related stigma, depression, and suicidal ideation among HIV-positive MSM in China, identifying depression as a mediator between HIV-related stigma and suicidal ideation, which underscores the significance of addressing depression in mitigating suicidal ideation across diverse populations. These studies collectively underscore the multifaceted role of depression in influencing suicidal ideation, mediated by various psychosocial and individual factors.

Since depression is a strong predictor of suicide, many studies have tried to elucidate these mechanisms [27,28]. However, only a few studies focused on depression and suicidal ideation [27,28]. Schmaal et al., (2020) [29] used imaging and reported that connectivity between various regions of the brain (e.g., frontal lobe, temporal lobe, limbic system, basal ganglia, posterior cingulate cortices, and hindbrain) was associated with suicidal ideation by reviewing the previous literature. Moreover, Kim et al., (2017) [30] used functional magnetic resonance imaging (fMRI) to find that depression decreased the function of the frontal lobe, which reduced the connectivity of the limbic system. They also emphasized that the decreased function of the frontal lobe made it difficult for the frontal lobe to control the limbic system, which further increased suicidal ideation by ultimately decreasing the connectivity of the limbic system [30]. Therefore, based on the results of these previous studies, depression was thought to have the most direct effect on suicidal ideation. It is necessary to conduct longitudinal studies that can reveal the causal relationship between depression and suicidal ideation in old age based on the results of this study.

It was found that relationship satisfaction with a child, among the variables related to social support, an environmental element, was a variable influencing the suicidal ideation of community-dwelling older adults. Recent studies have further explored the complex interplay between depression, social support, and suicidal ideation among older adults. For instance, Won et al., (2021) [31] investigated the mediating effect of life satisfaction on the relationship between depression and suicidal behavior among older adults, highlighting the importance of social support and life satisfaction in mitigating suicidal behavior. Similarly, Kim and Kihl (2021) [32] examined the role of social support in mediating the relationship between depression and suicidal ideation among older adults in the Republic of Korea, underscoring the potential of social support to diminish suicidal ideation by alleviating depression. In particular, our findings suggest that enhancing social support in the form of relationship satisfaction with children could be a vital strategy in reducing suicidal ideation among older adults. This result agreed with the results of previous studies, which reported that higher relationship satisfaction with a child decreased the frequency of suicidal ideation [33] and that relationship satisfaction with a child was related to emotional support in older adults [34,35]. Therefore, strategies that include older adults and their family relationships are needed to restore the social support of the community-dwelling elderly.

Another finding of this study was that IADL was a variable influencing suicidal ideation. The result was similar to the results of previous studies that examined the association between suicidal ideation and IADL [36,37]. Sim et al., (2021) [38] determined the effects of physical functioning on depressive symptoms and suicidal ideation among older Korean adults, finding that limitations to instrumental activities of daily living (IADL) were significantly associated with increased suicidal ideation. Specifically, compared to those with non-limited IADL function, individuals with limitations were more likely to exhibit depressive symptoms and suicidal ideation, highlighting the critical role of physical functioning in older adults’ mental health. Furthermore, Becattini-Oliveira et al., (2019) [38] assessed functional capacity in community-dwelling older adults and found a significant correlation between IADL performance and cognitive functions, suggesting that IADL assessments could serve as a screening tool for identifying older adults at risk of cognitive decline and associated mental health issues. These studies underscore the importance of addressing physical and functional capacities in mitigating mental health challenges among older adults.

IADL assesses the ability to perform activities of daily living, and difficulty with these activities indicates physical limitations. It has been reported that older adults with physical limitations have negative psychology such as low belongingness as a member of society and isolation [39], and participation in social activities can increase IADL scores [40]. Therefore, it is necessary to develop programs to increase IADLs for older adults with low IADL scores and carry them out so the participants can feel a sense of belongingness to the community within the developed programs.

This study offers fresh perspectives on the intricate relationship between depression, social support, and daily functioning, highlighting their collective significance in preventing suicidal ideation among the elderly. Despite these implications, this study had several limitations. First, the proportion of subjects with suicidal ideation was low in the raw data of this epidemiologic study. Second, the explanatory variables defined in this study were mainly measured by questionnaires. Future studies need to develop a suicidal ideation prediction model that includes medical records such as clinical data and disease history. Third, since this study used cross-sectional data, a temporal order of the variables was not established. In future, it will be necessary to analyze the causality according to the temporal order by using longitudinal data.

## 5. Conclusions

This study applied the suicidal ideation predictors of community-dwelling older adults to the ICF model to understand their effects on suicidal ideation and inferred the causal relationship using a path model. The results showed that gender, age, education level, economic level, the number of chronic diseases, depression, IADL, and relationship satisfaction with a child significantly affected suicidal ideation. It was confirmed that, among them, depression had the most direct effect on suicidal ideation and played a mediating role. It is necessary to develop community-level public health strategies based on these predictors. It is also required to further prove the causality of the variables identified as predictors from future longitudinal studies and check whether it is possible to generalize them to the entire older adult population.

## Figures and Tables

**Table 1:** General characteristics of study subjects.

Classification	Variables	Suicidal Ideation			
		Yes (*n* = 187)		No (*n* = 9733)	
		*n*	%	*n*	%
Impairments of body functions and structures	Subjective health status:				
	Poor	74	39.6	1786	18.3
	Average	61	32.6	3059	31.4
	Good	52	27.8	4888	50.2
	Depression:				
	No depression	57	49.5	8540	87.7
	Depression	100	53.5	1193	12.3
Personal Factors: socio-demographic factors	Gender:				
	Male	54	28.9	3917	40.2
	Female	133	71.1	5816	59.8
	Age:				
	65–74 years old	104	55.6	5873	60.3
	75–84 years old	78	41.7	3255	33.4
	≥85 years old	5	2.7	605	6.2
	Education Level:				
	Elementary school graduation or below	90	48.1	4341	44.6
	Middle school graduation	52	27.8	2278	23.4
	High school graduation	38	20.3	2616	26.9
	College graduation or above	7	3.7	498	5.1
	Household economic level:				
	National basic livelihood security recipient	37	19.8	688	7.1
	National basic livelihood security non-recipient	150	80.2	9045	92.9
Personal Factors: health factor	Chronic disease:				
	≤1	47	25.1	4553	46.8
	2–4	111	59.4	4725	48.5
	≥5	29	15.5	455	4.7
Environmental Factors	Formal support				
	(used a senior citizen center or senior welfare service center in the community in the past year):				
	Used	51	27.3	2907	29.9
	Never used	136	72.7	6826	70.1
	Informal support				
	(frequency of meeting an acquaintance in the past year):				
	≥2 times a year	42	22.5	1644	16.9
	1–2 times every 3 months	49	26.2	2749	28.2
	1–2 times a month	68	36.4	3761	38.6
	Once a week	18	9.6	1016	10.4
	Everyday	10	5.3	563	5.8
	Relationship satisfaction				
	(with children):				
	Dissatisfied	38	20.3	343	3.5
	Not satisfied or dissatisfied	53	28.3	2063	21.2
	Satisfied	96	51.3	7327	75.3
	Relationship satisfaction				
	(with friends/neighbors):				
	Dissatisfied	46	24.6	566	5.8
	Not satisfied or dissatisfied	64	34.2	3316	34.1
	Satisfied	77	41.2	5851	60.1
Activities	Instrumental Activities of Daily Living (IADL):				
	≤25 points	28	15	376	3.9
	≥26 points	159	85	9357	96.1
Social Participation	Employment status:				
	No	140	74.9	6166	63.4
	Yes	47	25.1	3567	36.6
	Participation in social groups:				
	No	137	73.3	5642	58
	Yes	50	26.7	4091	42

**Table 2:** Fitness of the predictive model.

CMIN/DF	GFI	AGFI	CFI	TLI	RMSEA	SRMR
2.811	1.00	0.995	0.999	0.989	0.014	0.004

**Table 3:** Standardized coefficients and multiple correlation coefficients of the predictive models.

Path		β	S.E.	C.R.	SMC
Subjective Health Status	← Education level	0.104	0.007	11.299 ***	0.338
	← Household economic level	0.033	0.025	3.918 ***	
	← Age	−0.094	0.012	−9.98 ***	
	← Chronic disease	−0.305	0.011	−35.411 ***	
	← IADL	0.143	0.033	16.634 ***	
	← Employment status	0.102	0.014	11.884 ***	
	← Informal support	0.083	0.014	9.149 ***	
	← Relationship satisfaction (with friends/neighbors)	0.131	0.012	14.234 ***	
	← Relationship satisfaction (with children)	0.067	0.013	7.565 ***	
Depression	← Subjective Health Status	−0.171	0.005	−15.688 ***	0.146
	← Household economic level	−0.053	0.012	−5.543 ***	
	← Chronic disease	0.101	0.006	9.789 ***	
	← IADL	−0.149	0.017	−15.187 ***	
	← Informal support	−0.031	0.003	−3.31 ***	
	← Relationship satisfaction (with friends/neighbors)	−0.068	0.006	−6.566 ***	
	← Relationship satisfaction (with children)	−0.074	0.006	−7.359 ***	
Suicidal ideation	← Gender	0.025	0.003	2.354 *	0.039
	← Education level	0.023	0.002	1.968 *	
	← Household economic level	−0.034	0.005	−3.401 ***	
	← Age	−0.03	0.003	−2.616 **	
	← IADL	−0.029	0.007	−2.707 **	
	← Employment status	−0.003	0.003	−0.325	
	← Participation in social groups	−0.016	0.003	−1.428	
	← Employment status	0.001	0.003	0.114	
	← Informal support	0.001	0.001	0.138	
	← Relationship satisfaction (with friends/neighbors)	−0.021	0.003	−1.846	
	← Relationship satisfaction (with children)	−0.066	0.003	−6.133 ***	
	← Subjective Health Status	0.002	0.002	0.144	
	← Depression	0.136	0.004	12.767 ***	
	← Chronic disease	0.028	0.003	2.504 *	

*: *p* < 0.05, **: *p* < 0.01, ***: *p* < 0.001.

**Table 4:** Effect decomposition results of the predictive model.

Path		Total Effect	Direct Effect	Indirect Effect
Subjective Health Status	← Education level	0.104 ***	0.104 ***	−
	← Household economic level	0.033 ***	0.033 ***	−
	← Age	−0.094 ***	−0.094 ***	−
	← Chronic disease	−0.305 ***	−0.305 ***	−
	← IADL	0.143 ***	0.143 ***	−
	← Employment status	0.102 ***	0.102 ***	−
	← Informal support	<0.001	<0.001	−
	←Relationship satisfaction (with friends/neighbors)	0.131 ***	0.131 ***	−
	← Relationship satisfaction (with children)	0.067 ***	0.067 ***	−
Depression	← Subjective Health Status	−0.171 ***	−0.171 ***	−
	← Household economic level	−0.058 ***	−0.053 ***	−0.006 ***
	← Chronic disease	0.154 ***	0.101 ***	0.052 ***
	← IADL	−0.174 ***	−0.149 ***	−0.023 ***
	← Informal support	−0.031 **	−0.031 **	−0.004
	←Relationship satisfaction (with friends/neighbors)	−0.091 ***	−0.068 ***	−0.022 ***
	← Relationship satisfaction (with children)	−0.086 ***	−0.074 ***	−0.011 ***
Suicidal ideation	← Gender	0.025 **	0.025 **	−
	← Education level	0.021	0.023 *	−0.002
	← Household economic level	−0.042 **	−0.034 **	−0.008 ***
	← Age	−0.028 **	−0.03 **	0.002
	← IADL	−0.052 **	−0.029 **	−0.023 ***
	← Employment status	−0.006	−0.003 ***	−0.002
	← Participation in social groups	−0.018	−0.016	−0.002
	← Employment status	0.001	0.001	<0.001
	← Informal support	−0.003	0.001	−0.004 **
	← Relationship satisfaction (with friends/neighbors)	−0.033 **	−0.021	−0.012 ***
	← Relationship satisfaction (with children)	−0.078 ***	−0.066 ***	−0.012 ***
	← Subjective Health Status	−0.022	0.002	−0.023 ***
	← Depression	0.136 ***	0.136 ***	<0.001
	← Chronic disease	0.048 ***	0.028 **	0.02 ***

*: *p* < 0.05, **: *p* < 0.01, ***: *p* < 0.001.

**Table 5:** Analysis results of individual indirect effect.

Path	Estimate
Subjective health status	
Education level → Subjective Health Status → Suicidal ideation	<0.001
Household economic level → Subjective Health Status → Suicidal ideation	<0.001
Age → Subjective Health Status → Suicidal ideation	<0.001
Chronic disease → Subjective Health Status → Suicidal ideation	−0.001
IADL → Subjective Health Status → Suicidal ideation	<0.001
Employment status → Subjective Health Status → Suicidal ideation	<0.001
Participation in social groups → Subjective Health Status → Suicidal ideation	<0.001
Relationship satisfaction (with friends/neighbors) → Subjective Health Status → Suicidal ideation	<0.001
Relationship satisfaction (with children) → Subjective Health Status → Suicidal ideation	<0.001
Depression	
Household economic level → Depression → Suicidal ideation	−0.007 ***
Chronic disease → Depression → Suicidal ideation	0.014 ***
IADL → Depression → Suicidal ideation	−0.02 ***
Informal support → Depression → Suicidal ideation	−0.004 ***
Relationship satisfaction (with friends/neighbors) → Depression → Suicidal ideation	−0.009 ***
Relationship satisfaction (with children) → Depression → Suicidal ideation	−0.001 ***
Subjective health status → Depression	
Education level → Subjective Health Status → Depression → Suicidal ideation	−0.002 ***
Household economic level → Subjective Health Status → Depression → Suicidal ideation	−0.001 ***
Age → Subjective Health Status → Depression → Suicidal ideation	0.002 ***
Chronic disease → Subjective Health Status → Depression → Suicidal ideation	0.007 ***
IADL → Subjective Health Status → Depression → Suicidal ideation	−0.003 ***
Employment status → Subjective Health Status → Depression → Suicidal ideation	−0.002 ***
Participation in social groups → Subjective Health Status → Depression → Suicidal ideation	−0.002 ***
Relationship satisfaction (with friends/neighbors) → Subjective Health Status → Depression → Suicidal ideation	−0.003 ***
Relationship satisfaction (with children) → Subjective Health Status → Depression → Suicidal ideation	−0.002 ***

***: *p* < 0.001.

## Data Availability

Data are contained within the article.
